# Assessment of Influencing Factors and Robustness of Computable Image Texture Features in Digital Images

**DOI:** 10.3390/tomography11080087

**Published:** 2025-07-31

**Authors:** Diego Andrade, Howard C. Gifford, Mini Das

**Affiliations:** 1 Department of Biomedical Engineering, University of Houston, 4800 Calhoun Rd, Houston, TX 77004, USA; dfandrade@uh.edu (D.A.); hgifford@uh.edu (H.C.G.); 2 Department of Electrical and Computer Engineering, University of Houston, 4800 Calhoun Rd, Houston, TX 77004, USA; 3 Department of Physics, University of Houston, 4800 Calhoun Rd, Houston, TX 77004, USA

**Keywords:** breast imaging, clinical and simulated data, density, digital breast tomosynthesis (DBT), gray level co-occurrence matrix (GLCM), partial angle, radiomics and texture features

## Abstract

**Background/Objectives:** There is significant interest in using texture features to extract hidden image-based information. In medical imaging applications using radiomics, AI, or personalized medicine, the quest is to extract patient or disease specific information while being insensitive to other system or processing variables. While we use digital breast tomosynthesis (DBT) to show these effects, our results would be generally applicable to a wider range of other imaging modalities and applications. **Methods:** We examine factors in texture estimation methods, such as quantization, pixel distance offset, and region of interest (ROI) size, that influence the magnitudes of these readily computable and widely used image texture features (specifically Haralick’s gray level co-occurrence matrix (GLCM) textural features). **Results:** Our results indicate that quantization is the most influential of these parameters, as it controls the size of the GLCM and range of values. We propose a new multi-resolution normalization (by either fixing ROI size or pixel offset) that can significantly reduce quantization magnitude disparities. We show reduction in mean differences in feature values by orders of magnitude; for example, reducing it to 7.34% between quantizations of 8–128, while preserving trends. **Conclusions:** When combining images from multiple vendors in a common analysis, large variations in texture magnitudes can arise due to differences in post-processing methods like filters. We show that significant changes in GLCM magnitude variations may arise simply due to the filter type or strength. These trends can also vary based on estimation variables (like offset distance or ROI) that can further complicate analysis and robustness. We show pathways to reduce sensitivity to such variations due to estimation methods while increasing the desired sensitivity to patient-specific information such as breast density. Finally, we show that our results obtained from simulated DBT images are consistent with what we see when applied to clinical DBT images.

## 1. Introduction

Technological advancements have paved the way for significant progress in quantitative imaging and introduced novel data-centered analytic methods. Texture analysis holds significant importance in various imaging fields due to its ability to provide statistical, structural, and intrinsic spatial information from images. It enables us to identify and characterize objects based on their unique texture signature. Extensive research has been dedicated to comprehending and quantifying these texture features, with contributions from prominent researchers such as Haralick, Amadasun, Julesz, Galloway, and others [1,2,3,4,5]. They have been applied in agriculture [6,7], topography [8,9], computer vision [10,11], astronomy [12,13], and many other areas. Their second and higher-order texture characterization methods employ statistical information to decode valuable image information, and quantitatively represent it.

One of the most common and widely used approach is Haralick’s gray level co-occurrence matrix (GLCM) [2]. This matrix allows visualization of the frequency at which various combinations of pixel gray levels or brightness occur in the image of interest. The repetitions can be examined for pixels across an image at a certain angle and chosen distance (offset) between them. To be computationally effective, the entire image is divided into regions of interest (ROI)/volumes of interest (VOI) and a GLCM is estimated for each ROI. Relevant texture features are calculated from GLCM.

Texture features can be calculated either globally, using the entire image, or locally within specific ROI or VOI relevant to the task at hand. The order of these features depends on the number of pixels involved in their calculation, which means that a texture could be intra- or inter-pixel calculated. First-order features, also known as histogram-based features (such as mean, standard deviation, kurtosis, and skewness), are calculated using individual pixel values. However, first-order features provide limited structural and spatial information, making it essential to explore second- and higher-order texture features. These higher-order features provide additional insights by characterizing the interaction of nearby pixels at specific distances and angles.

In medical imaging, researchers have incorporated GLCM texture features with radiomics, and in both supervised and unsupervised learning models. They have been used in tasks such as tissue segmentation [14,15], lesions classification [16,17,18,19], disease prognosis, progression, and risk assessment [20,21,22,23,24,25,26,27]. The variations in estimated texture magnitudes due to the wide selection of parameters, processing techniques, and imaging physics has led to questions about the reliability of these approaches. Mao et al. [28], Chaddad et al. [29], Hwan-ho Cho and Hyunjin Park [30], and others, have demonstrated the wide scope of calculation parameters such as number of angles, pixels offsets, ROI sizes, and quantization levels under which texture features can be obtained. Researchers like Robins et al. [31] in CT simulated phantoms, Keller et al. [32] in simulated full-field digital mammography (FFDM) on different imaging vendors, and others have explored the variability and reliability of these features across different acquisition settings, spatial configurations, quantization levels, interpolations, and processing methodologies [33,34,35,36,37,38,39,40]. Foy et al. [41] explored software packages for GLCM features calculations, finding variability even in the default parameters and processing techniques. Their studies highlight the importance of establishing reliability and further investigations into texture parameters and calculations, contributing to a more comprehensive understanding of texture-based analysis in diverse imaging applications and modalities. Our group recently proposed the potential of utilizing texture features to predict human observer detection performance in digital images and to assess the influence of anatomical and quantum noise on their origins [42,43,44,45,46].

Despite the potential of radiomics, concerns about robustness and generalization remain. For instance, in digital breast tomosynthesis (DBT) alone, for applications like lesion discrimination [47,48,49,50,51], risk assessment [52,53], and prognosis models [54,55,56,57], even when similar approaches and texture feature libraries are used, different features are often selected in the final models. This variability can be attributed to factors such as lesion heterogeneity, exclusive focus on tumoral regions, and differences in grayscale level selection. As a result, ROI size and quantization parameters appear as highly important factors, contributing to the variation in outcomes across similar models.

An in-depth and systematic examination on the robustness of GLCM features, based on the combination of pre-processing, acquisition techniques, and calculation parameters has not been previously investigated. This work aims to fill this gap by examining the magnitude variability of GLCM features with variables involved in GLCM texture estimation. We will do so using DBT as a modality of choice using both simulated and clinical data. We use DBT images simulated under the exact same conditions, including acquisition and reconstruction methods. For these simulated images, we examined variations in magnitudes of estimated texture features due to changes in estimation parameters (like quantization, ROI, and offset). We assessed how other aspects such as post-processing methods like filtration could add to complexity in assessing texture magnitudes. Variations in post-processing methods and filtrations are common when data from multiple vendors systems are combined in applications involving AI or radiomics. Separately, we also examined these texture variations when breast phantoms of different densities are imaged. Our goal here is to understand if there are texture features or texture estimation methods which are more sensitive to patient-specific information (like breast density) while being insensitive to estimation methods when the same system is used for acquisition. Working with realistic and simulated images allow such in-depth analysis.

The use of simulated data also offers advantages such as prior knowledge of ground truth, ability to control acquisition parameters, breast density, and reconstruction methodologies to test our premises. Finally, comparisons with clinical DBT images allowed us to assess the reliability and applicability of our findings from simulated DBT images to practical applications with clinically acquired images.

Finally, in this manuscript, we consider robustness of a texture feature as the ability to maintain consistent and reliable values despite variations in imaging conditions or variables involved in texture calculations. This can be understood by analyzing trends of the features, and identifying features whose magnitudes flatten or increase/decrease as the parameters used in texture calculations change. In this work, we also examine.

## 2. Materials and Methods

DBT is a partial angle tomographic breast imaging system. It acquires a limited number of projections of the breast at various angles along a selected arc span. Subsequently, these 2D projections/slices are used to reconstruct a pseudo 3D volume of the breast. Unlike a conventional mammogram, which provides a 2D view, DBT images offer radiologists a 3D view with thin-sliced images (typically around 1 mm slice thickness) of the breast.

**Figure 1:**
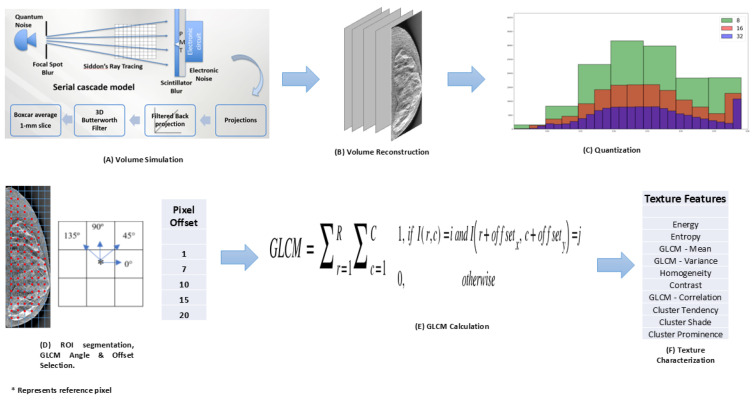
Workflow for texture analysis of simulated DBT images. The workflow consists of six stages: (**A**) volume simulation, (**B**) volume reconstruction from simulated projections, (**C**) quantization of reconstructed volumes, (**D**) ROI segmentation, GLCM angle, and offset selection, (**E**) GLCM calculation, and (**F**) texture features calculation.

The framework for our analysis includes a simulation platform (Figure 1A), volume/image reconstruction (Figure 1B), application of different quantization levels to our images (Figure 1C), selection of GLCM calculation parameters (Figure 1D), GLCM calculation for the different parameters combinations (Figure 1E), and GLCM texture features quantification (Figure 1F). Leveraging prior knowledge of ground truth related to the phantom and the system provided the required flexibility to test our premises under various simulated scenarios. From step Figure 1C forward, we used an in-house Python 3.9 script to perform our image processing and textures’ calculations. The simulation platform employed has been described in several of our prior publications. However, we give a brief summary here for completeness.

### 2.1. Volume Simulation

The computer simulation platform has been developed and tested to examine several questions on imaging dose vs. detectability of breast mass and microcalcifications [58,59,60,61,62]. An ensemble of realistic breast phantoms generated by Bakic et al. [63] and phantoms with two different breast densities (25% and 50%) were used for our study. Breast density refers to the proportion of fibrous and glandular tissue in comparison to adipose in the breast. The digital phantoms used in this study have a uniform thickness of 5 cm similar to the average clinical compressed breast reported by Hendrick et al. [64]. The simulation platform allows generation of image sets with a range of acquisition and processing strategies with physical models of imaging system gains and noise [65]. More specifically, strategies such as an X-ray source with tungsten anode and a CsI detector with 0.1 mm thickness based on aSi:H flat panel detector. Siddon’s ray-tracing algorithm is applied to model X-ray transmission through the voxelized phantom with voxel dimensions of 0.2 mm × 0.2 mm × 0.2 mm. In this process the source spectrum, X-ray projection through digitized 3D object phantoms, detector gain, noise, and system blur are characterized as well. Quantum detection and optical collection efficiency, and scintillator gain are also taken into consideration. Noise propagation through the detector was modeled as a scaled Poisson process along with an additive electronic noise.

### 2.2. Volume Reconstruction

**Figure 2:**
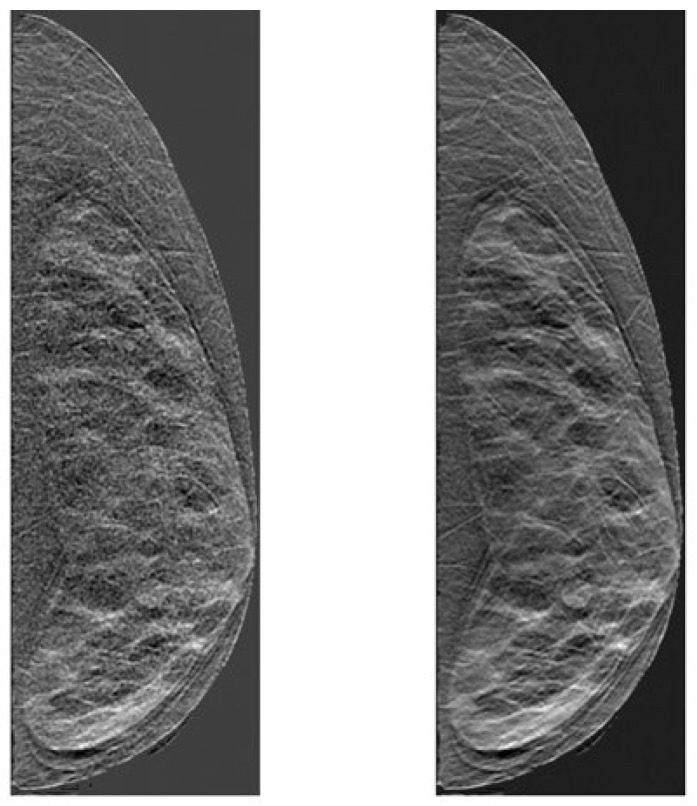
An example of simulated DBT slice, (**left**) without and (**right**) with Wiener filter applied to the reconstructed volume. These images correspond to a digital breast phantom with 25% breast density.

The simulated DBT projections were reconstructed to obtain a pseudo 3D volume in DBTs. The reconstruction process involves utilizing Feldkamp-filtered back projection and applying a 3D Butterworth filter with a cut-off frequency of 0.25 cycles/pixel. An adaptive Wiener filter [66] was applied to the noisy projections due to its efficiency in reducing noise without structural deterioration in this imaging modality. The Wiener filter [66] approximates the noise present in the images to a Gaussian additive noise, and reduces it by minimizing the mean square error. The reconstructed image slices are of 0.2 mm thickness and have a dimension of 760 × 240 with in-plane resolution of 0.27 × 0.27 mm; to mimic clinical settings they are slabbed to 1 mm thick slices for further texture analysis. Both images with and without the Wiener filter are subsequently used in our analysis to assess the effects of image filtering in texture features. An example of a simulated DBT image slab is shown in Figure 2.

### 2.3. Clinical Data

**Figure 3:**
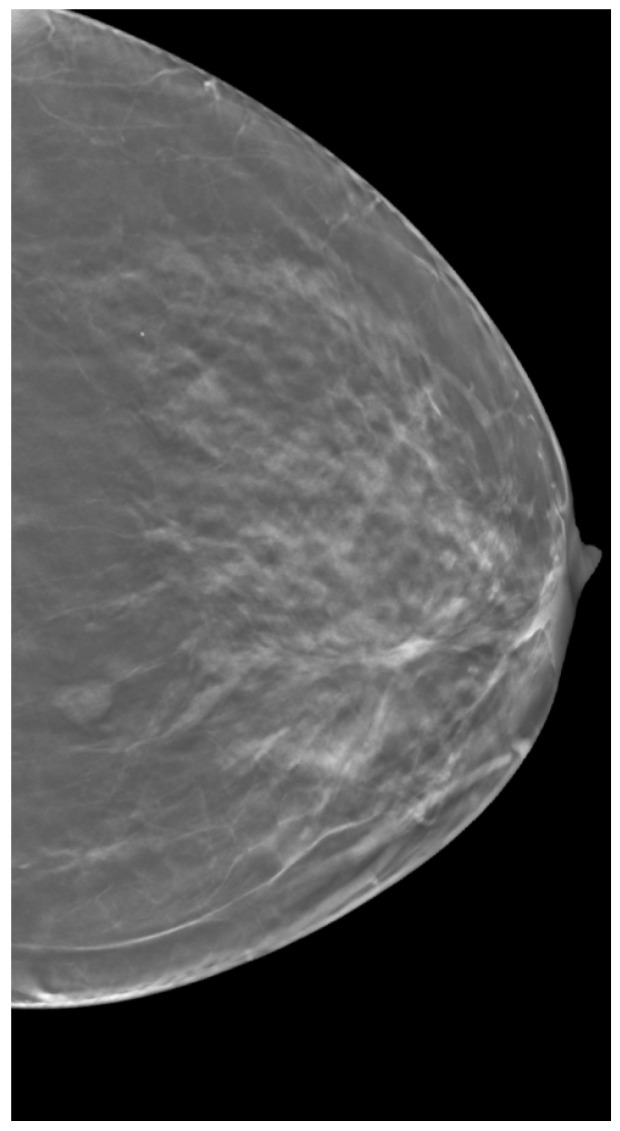
An example of DBT slice from clinical dataset obtained from The Cancer Imaging Archive.

The dataset was obtained from The Cancer Imaging Archive, which is funded by the Cancer Imaging Program, a part of the United States National Cancer Institute, and is managed by the Frederick National Laboratory for Cancer Research [67]. This curated dataset comprises DBT images of 5060 subjects, including cases of normal breast tissue, actionable biopsy-proven benign, and biopsy-proven cancer cases [68]. The DBT images were acquired using Selenia Dimensions from Hologic, with an arc span of 15° and 15 projections. For our analysis, we focused on a subset of 90 DBT-labeled images containing both benign and cancerous lesions. A clinical case example used in our analysis is shown in Figure 3. Calculations were conducted in lesion-free regions of the images to maintain consistency with our simulated dataset. These calculations were also performed under comparable parameters combinations as used in the simulated data.

### 2.4. Texture Calculations

#### 2.4.1. Quantization

**Figure 4:**
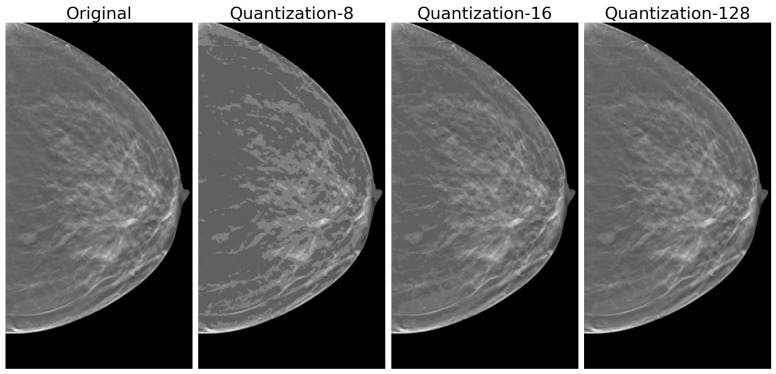
Visual comparison of quantization effects on DBT clinical image. Three different quantization levels from **left** to **right**: original image, 8, 16, and 128 gray levels. At 8 gray levels, significant artifacts are visible with loss of overall quality and elements representation. The 16-level quantization shows improved tissue representation while still exhibiting some contouring effects. The 128-level quantization closely approximates the original image, preserving fine details. This comparison illustrates the trade-off between computational efficiency (lower quantization levels) and preservation of image quality in texture analysis applications, highlighting the importance of selecting appropriate quantization parameters for clinical imaging studies.

In the context of image processing, quantization entails grouping pixel values into fixed-size or a fixed-number of bins, in our case, of grayscale levels. The quantization level must be chosen carefully, striking a balance between representing all tissues and textures present in the image and avoiding information loss. As mentioned in [35], higher quantization levels may represent noise and make homogeneous volumes appear heterogeneous. Conversely, low quantization levels may lead to the loss of relevant textural information, producing an adverse effect on the displayed image. This effect can be seen in Figure 4, in which at 8 gray levels, loss of overall quality and elements representation are visible. As quantization increases we observe improvements in quality, and at 128 levels, the quantized image approximates the original with a visible preservation of fine details. Although the quantization level is not a direct calculation parameter of the textures, it can significantly impact the magnitudes, complexity, and computational resources as it directly dictates the size of the GLCM as illustrated in the “GLCM Calculation” section below.

For our study, we examine four quantization levels: 8, 32, 64, and 128. The latter three levels are commonly employed in similar experiments due to their ability to maintain proper visual fidelity in medical images. The image biomarker standardization initiative (IBSI), also suggests the usage of 32 and 64 when extracting second- and higher-order texture features. To prepare the images for quantization, we performed min-max normalization scaling the data to the range [0, 1], and then multiplied the results by the selected quantization. This process allows us to bin the values in the desired number of grayscale levels. The resulting values were rounded to the nearest integer representing the gray level bin, as second-order texture features require integer values for their calculations. In our Equation (1), ‘*I*’ denotes the image, and ‘*Q*’ represents the selected quantization level.

(1) $$I_{Quantized}=\frac{I_{PixelValue}-I_{Min}}{I_{Max}-I_{Min}}*Q_{Level}$$

#### 2.4.2. ROI Segmentation, GLCM Angle, and Offset Selection

The ROI is defined as a multi-pixel neighborhood of analysis, within which desired calculations are performed. Within the neighborhood of pixels, three essential characteristics, namely brightness difference, pixel offset, and directionality, play a crucial role in the texture calculations, all of which are considered by the GLCM features. To evaluate the texture features, textures are calculated on symmetrical non-overlapping square ROIs, as shown in Figure 1E. In our analysis, we employed squared ROIs with sizes of 10, 25, 50, and 100 ${\mathrm{pixels}}^{2}$. These size selections were based on our images’ dimensions and internal components, ensuring that within these ranges, the different structures present in our images are properly characterized. For our simulated images, a lattice-based approach was taken to subdivide the entire breast into equisized ROIs as illustrated in Figure 1D. For clinical images, the selected locations were outside of the lesion limits identified in the dataset files provided. This was to ensure that lesions heterogeneity did not affect the analysis. It then was subdivided to match the ROI sizes similar to those selected in analysis of our simulated images. We use the lesions/signal as a center of reference for the slice selection in both simulated and clinical images to ensure spatial consistency.

**Figure 5:**
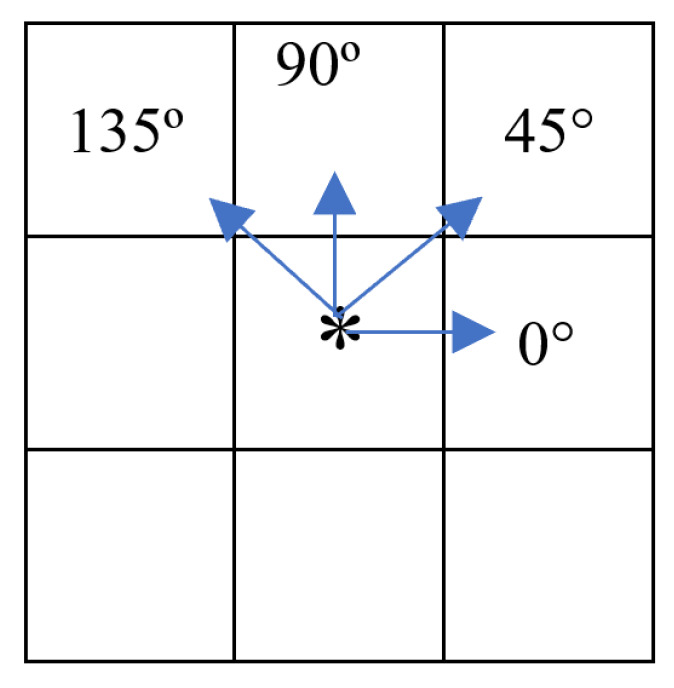
GLCM pixels direction comparison and possible calculation angles. The asterisk symbolizes the main pixel (*i*) and the blue arrows angular direction of possible secondary pixel selection (*j*).

Once the areas of interest are segmented, we followed by combining a selection of angles and pixel offsets within the different ROI sizes for the GLCM calculation. In our study, Figure 5 illustrates the eight possible adjacent pixels surrounding the principal pixel at different angles. However, for efficiency, only four of these are explicitly calculated, namely 0°, 45°, 90°, and 135°, as the opposite directions (+180°) are equivalent to their respective GLCM transposes. In our literature survey, we observed that the GLCM angular directions are assumed to be isometric. This implies that equal weight is assigned to the calculated textures regardless of the angular direction used for calculation. We proceeded in our research under the same assumption, and average the magnitudes among angles. Regarding pixel offset, the default distance between pixels used for GLCM calculations is typically set to one and referred as *d*; it is worth noting that this value can range from one to the size of the image minus one. For the offset selection we performed our calculations using 1, 5, 7, 10, 15, and 20 pixels. Based on our investigation, we observed that models have not typically exceeded a 5-pixel offset. However, our results indicated a lack of robustness within this range, prompting us to increase the range for better understanding.

#### 2.4.3. GLCM Calculation

**Figure 6:**
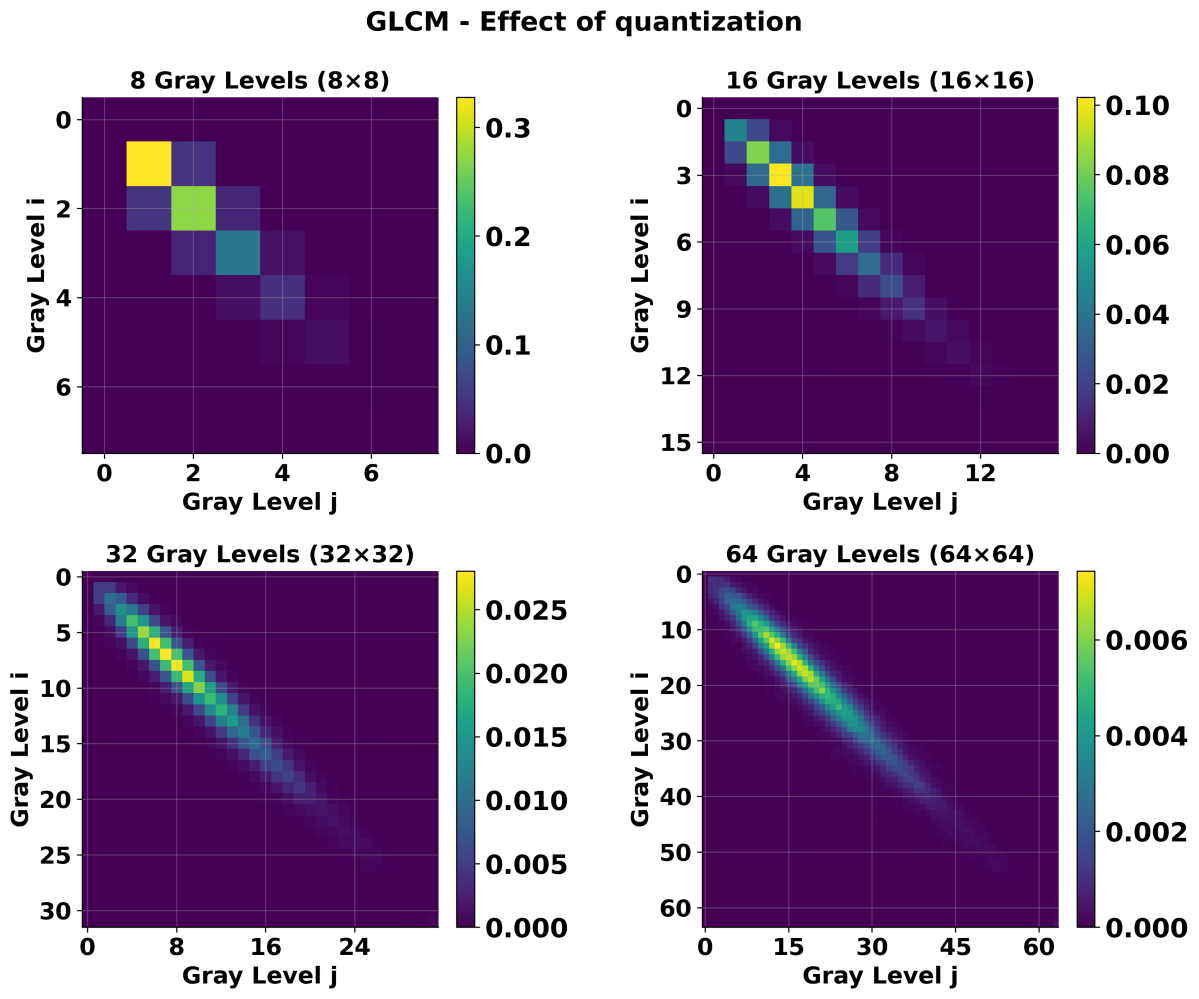
Effect of quantization on GLCM size and probability distribution represented by G(i,j). Heatmaps showing GLCMs computed from the same image using different quantization levels: 8, 16, 32, and 64 gray levels, resulting in corresponding matrix dimensions of 8 × 8, 16 × 16, 32 × 32, and 64 × 64. The color scale represents co-occurrence probabilities, with brighter regions indicating higher probability values. Lower quantization levels (8, 16) produce concentrated probability distributions with higher maximum values, while higher quantization levels (32, 64) result in more dispersed, diagonal-dominant patterns with lower individual probabilities due to increased matrix sparsity.

We performed the GLCM calculation using our four selected quantization levels in combination with our defined ROIs and pixel offsets. For this task, we count the frequency of pairs of pixel repetitions (i,j), with ‘*i*’ being the reference pixel and ‘*j*’ being the pair at a certain orientation angle θ and distance offset *d* from ‘*i*’. In the next step, the GLCM pixel pair counts are scaled between [0, 1], by dividing them by the sum of all counts. This scaling allows us to obtain the “probability” of occurrence of pixel pairs with intensities (i,j) within the image represented as G(i,j). Figure 6 is a visual representation of the effects of quantization in the GLCM. It shows GLCMs of an image quantized at 8, 16, 32, and 64 gray levels, resulting in corresponding matrix dimensions of 8 × 8, 16 × 16, 32 × 32, and 64 × 64. As quantization levels increase, enabling more possible pixel pair combinations, the G(i,j) distributions shift from concentrated high-value peaks to more (diagonally) dispersed patterns with lower individual probabilities. After computing the GLCMs for the specified directions, texture features can be computed and defined.

#### 2.4.4. Textures Calculations

GLCM texture-based features are statistical characterizations designed by Haralick to quantitatively measure different characteristics between pairs of pixels. These features are specifically designed to quantify various types of textures present in the image. According to Hall-Beyer [8], GLCM features can be grouped into three categories: order, contrast/structural, and descriptive statistical features. Additionally, we introduce a fourth category called cluster features. Order features measure the regularity of pixel pair appearances. For example, energy increases as a single pair of pixels appears more frequently in the ROI, providing order/uniformity. Structure features assess the distance between grayscale levels of pixel pairs. As examples in this category, contrast increases with greater distance between pairs, while homogeneity decreases. Descriptive statistical features calculate common statistics from the GLCM itself, not from the original image. Finally, cluster features focus on the skewness, asymmetry, uniformity, and likelihood of cluster formations. The selected features for our analysis are listed in Table 1, along with their respective formulas.

In these formulas:

- G(i,j) represents the probability of the pair of pixels (i,j) in the image.
- θ denotes the angle at which the GLCM is calculated.
- *d* represents the pixel distance between the pixels (i,j).
- μ represents the mean, and σ corresponds to the standard deviation.

Calculations are performed for each ROI window size across the image at the four previously mentioned angular directions. To obtain the texture magnitude, we average each parameters’ combination across the image.

## 3. Results

We begin by examining the effects of the Wiener filter on our images to assess the variability that a widely used noise reduction filter has on texture magnitudes. We then proceed to analyze the effects of quantization to measure the magnitude changes due to data binning. Finally, we focus on the effect of anatomical differences using different simulated breast densities. All of the above were also calculated using different ROI sizes and pixel distance offsets to examine how they influenced each other.

### 3.1. Filtering

**Figure 7:**
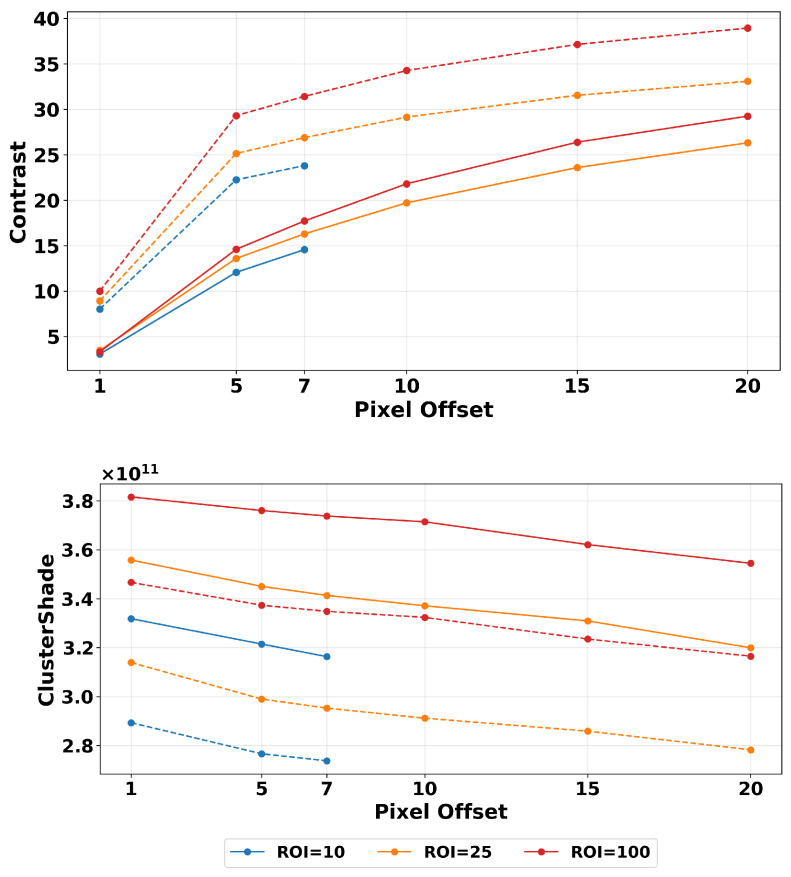
Magnitude variation of two texture features (contrast and cluster shape) from simulated DBT images with (solid line) and without (dashed line) Wiener filtering. Results shown over different distance offsets and ROI sizes with a quantization of 32 grayscale levels. Filtering reduces sharp changes seen with smaller offsets. We see also improved robustness with larger ROIs used in texture estimation.

We first examined how the changes in magnitude relate to image filtering based on ROI size and pixel distance variations. In Figure 7 the color lines represent different ROI sizes, the x-axis represents pixel offsets, and the y-axis represents texture magnitude. It shows the results of two texture features, contrast and cluster shade, showcasing representative variations found across our features. To investigate the impact of filtering, we performed our calculations on images before and after applying Wiener filtering. From Figure 7, we can observe that applying the filter resulted in smoother transitions between points in closer offset selections, reducing variability. This is important because, as we mentioned before, most literature focuses on short offset selection. For example, contrast starts exhibiting higher robustness beyond a distance offset of five pixels as the slope steepness is reduced.

Analyzing the contrast feature plot, we observe that the lines for filtered images are closer together. This is understood as filtering reducing the effect of ROI size selection, making it more robust to the area of calculation. A similar effect is present in the other structural features, shown in more detail below in the Normalization section heat map. The plots showing changes in estimated contrast texture features with changing pixel offsets appear grouped together when examining changes in ROI sizes. This shows that ROI size selection itself did not significantly influence values here as much as the change due to the image being filtered vs unfiltered. The magnitude difference between images with and without Wiener filter is very similar among the ROI sizes as seen in Table 2. These results show the average and percentage magnitude difference of consecutive pixel offsets beyond five pixels. We focus on this area as it is where textures display more robustness compared to smaller values.

On the other hand, beyond the pixel offset value of seven, the cluster shade demonstrates relatively high robustness. In this case, we see the average percentage change is very low within each ROI. The ROI of 100 ${\mathrm{pixels}}^{2}$ displays the highest magnitude difference between images with and without Wiener filter, with the exception of filtered cluster shade. This indicates that pixel offset influences less on the variability of this feature than ROI size. This is present in images with and without the Wiener filter. In the contrast feature, we see that the values are smaller in the filtered dataset. This can be attributed to how the Wiener filter works. In its initial step an averaging kernel (low-pass filter) is executed on the input image [66], which makes the pixel differences in the affected area smaller, resulting in lower feature values, as shown in our results. Conversely, the cluster shade shows increased magnitude in the Wiener-filtered images compared to unfiltered ones. This increase occurs because the Wiener filter reduces noise in the image, which also reduces the overall image mean. This reduction lowers both $\mu_{x}$ and $\mu_{y}$ values in the GLCM, resulting in larger values for ${\left(i+j-\mu_{x}-\mu_{y}\right)}^{3}$ (See Table 1). As a consequence, it results in higher cluster shade magnitude. This effect can be visually observed in Figure 2, where asymmetrical patterns are more apparent in the Wiener-filtered images.

### 3.2. Quantization Effects

**Figure 8:**
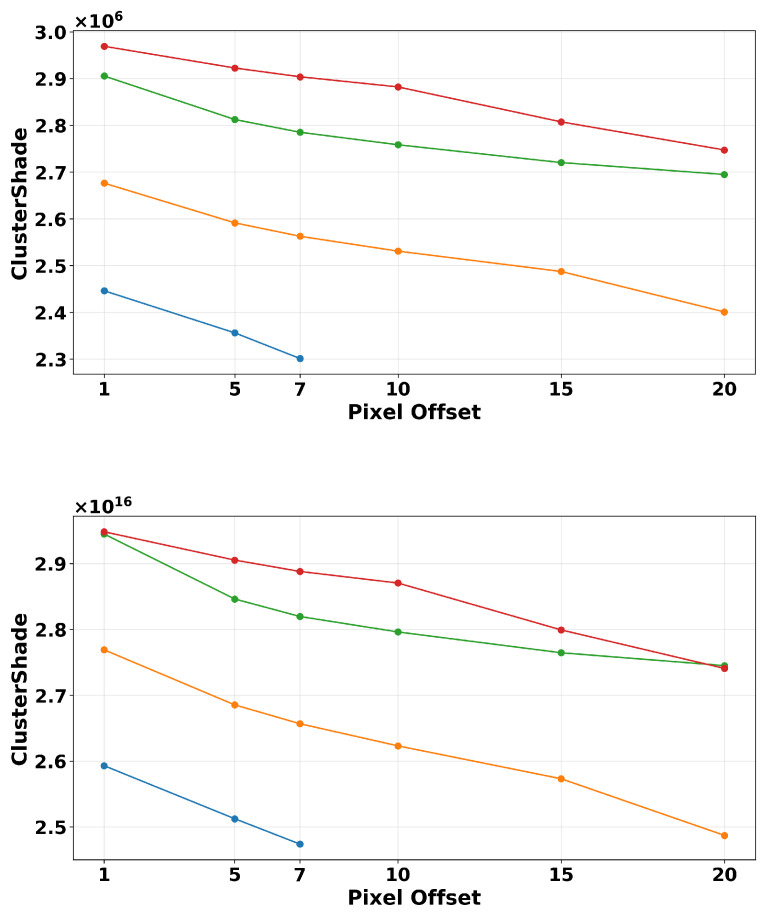
Texture magnitudes on simulated filtered images calculated under different quantization Levels. (**Top**): Cluster shade 8-grayscale levels. (**Bottom**): Cluster shade 128-grayscale levels. These graphs’ y-axis, which is the magnitude value of the texture, display the significant magnitude change between these two quantization levels. This can cause a great disparity in the results of a model if used as inputs/predictors.

The size and complexity of the GLCM is dependent on the image quantization level, making it a crucial characteristic to be considered in texture analysis. To highlight the variability produced by quantization, we display our results using the lowest and highest levels we calculated on. Figure 8 shows how quantization level significantly affects the magnitude of texture metrics. Higher quantization levels result in larger magnitude values for both contrast and cluster shade. Table 3 illustrates, numerically, the differences between these two quantization levels for both textures. It displays the minimum and maximum values obtained among all the images in the dataset to emphasize the range of values from which the means in the plot are calculated and the high variability between quantization levels. Despite the high variations in magnitude, the trends remain quite similar. This analysis aligns with the results obtained in [35] on PET imaging modality, which displays similar variation trends with varying quantization levels. Both of these features are characterized as functions of exponential nature, causing their magnitudes to be rapidly affected by the chosen quantization level.

### 3.3. Breast Density

**Figure 9:**
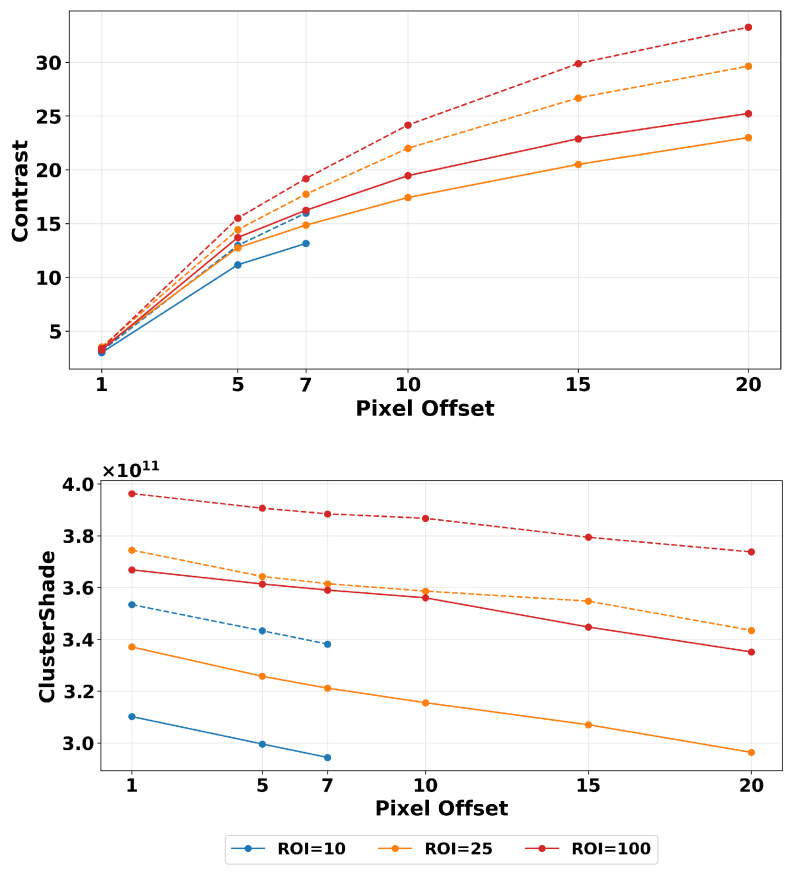
Texture magnitudes on simulated filtered images for different breast densities, 25% represented by dashed and 50% solid lines. These graphs highlight how physiological differences affect texture magnitudes and using smaller ROIs reduce these effects. Cluster shade shows good separation between densities across all the scenarios, while contrast increases differentiation as the offset increases.

**Figure 10:**
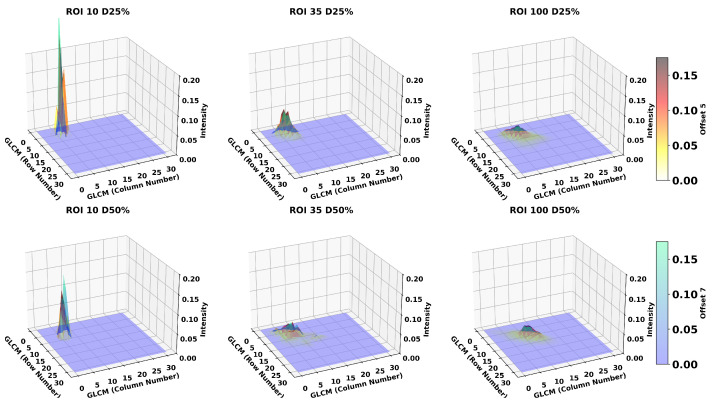
GLCM surface plots showing the effect of ROI size and density. Surface plots display G(i,j) distributions for ROI sizes of 10, 35, and 100 pixels at densities of 25% (**top row**) and 50% (**bottom row**), calculated using offsets of five (yellow-red) and seven (blue-green) pixels as indicated in the color bars. The x- and y-axes represent GLCM column and row numbers respectively, while the z-axis (intensity) indicates the probability of co-occurrence values for both offsets. Smaller ROIs (**left column**) exhibit sharp, localized peaks indicating high sensitivity to local variations, while larger ROIs (**right column**) show broader, more distributed patterns demonstrating increased robustness and stability in cluster texture measurements.

Finally, while just a patient characteristic and not a user introduced variable in texture estimation, we conducted our analysis by examining the effects of different breast densities on the magnitudes of textures. Leveraging our realistic simulation framework, we had the capability to modify breast density, enabling a better understanding of its impact on texture magnitudes. In Figure 9, we present the results on 25% and 50% breast densities. Notably, using smaller ROIs better mitigates the effects caused by increased density. A lower breast density leads to lower values of both contrast and cluster shade, particularly when the ROI size is larger. As observed in Table 4, a higher-density cluster shade shows increased robustness as the ROI increases. This effect can be more clearly observed in the cluster shade results for ROIs of size 100. Contrast is more robust at lower densities, provided that one tissue predominates over the other, in this case adipose. Cluster shade on the other hand is more robust in higher densities, specially as the ROI size increases. This effect can be explained more clearly in Figure 10. We observe that in larger ROIs, kurtosis of the GLCM matrix distributions are lower compared to smaller ROIs, increasing the likelihood of more clusters forming away from the mean, but make it less likely to change based on the offset as shown by the GLCMs. It should be mentioned that for applications requiring density differentiation, our simulated images demonstrate that this could be achieved using either contrast features with larger ROIs and pixel offsets, or cluster shade features regardless of the specific parameters chosen.

As demonstrated in this and the preceding subsections, all the tested parameters influence the magnitude changes in the texture features, which raises concerns about their application as radiomic metrics or inputs in AI models, particularly when used as single-value magnitudes. However, the observed similarities in the texture trends offer promising results. Based on these, a mathematical constant factor could be used to compensate for the variations caused by these parameters. To investigate this possibility, especially in the context of quantization changes, we explore the application of normalization on the texture features in the next subsection.

### 3.4. MANOVA

We conducted a statistical analysis to assess the robustness of Haralick texture features to parameter variations using multivariate analysis of variance (MANOVA) and univariate ANOVA with interaction terms on six texture features after averaging the correlation filtering across all scenarios (threshold > 0.85). As seen in Table 5, all texture features demonstrated significant sensitivity to the three experimental parameters (*p* < 0.001 for all main effects). The explained variance ($R^{2}$) ranged from 75.9% (contrast) to 98.7% (correlation), indicating strong parameter influence across all features.

The inclusion of two-way interaction terms improved model performance, with $R^{2}$ values increasing to 91.7–99.8% (Table 6). All features exhibited significant interactions for ROI × distance and ROI × quantization. For distance × quantization, all interactions were significant except for the texture Mean, indicating complex dependencies that cannot be captured by analyzing one variable at a time.

The full interaction model revealed significant three-way interactions (ROI × distance × quantization) for all texture features (*p* < 0.001), with F-values ranging from 4.46 (mean) to 580.27 (contrast). This finding indicates that the combined effect of all three parameters creates unique patterns that differ from the sum of their individual and pairwise effects.

Our findings show that Haralick texture features exhibit sensitivity to extraction parameters, with significant interactions indicating that parameter effects are not independent. The presence of three-way interactions for all features suggests the following:

1. Parameter optimization cannot be performed independently for each factor.
2. Texture analysis protocols should specify all three parameters.
3. Cross-study comparisons require identical parameter configurations.
4. Feature robustness varies significantly with the specific parameters combination used.

The high $R^{2}$ values (75.9–99.8%) indicate that parameter selection accounts for the majority of variance in texture measurements, highlighting the importance of standardized calculation protocols for reproducible texture analysis.

### 3.5. Multi-Resolution Normalization

Normalization is a widely used approach in data analysis to address differences in variables ranges, such as those observed in GLCM texture features calculated under different scenarios. To provide a more consistent and robust metric when there are multiple variables, we propose a multi-resolution min-max normalization. We show how it could mitigate the effect of texture variability due to changes in quantization used. Here, for each ROI, we use the mean texture values obtained at different pixel-offset distances and normalize across them to the [0, 1] range. This approach prevents outliers from distorting features trends of the selected ROI, which is a risk of normalizing each combination independently. Alternatively, this process can be performed by focusing on each distance offset and using the ROIs’ means for normalization, yielding similar results with respect to quantization effects. This operation is applied across all ROIs for each quantization level independently.

(2) $$Tex_{normalized}=\frac{Tex_{value_{d}}-Tex_{ROI_{min}}}{Tex_{ROI_{max}}-Tex_{ROI_{min}}}$$

The term $Tex_{value_{d}}$ refers to the texture magnitude of an ROI size at a specific distance offset. $Tex_{ROI_{min}}$ and $Tex_{ROI_{max}}$ represent the minimum and maximum texture magnitude values, considering all the offsets of that ROI window size. This approach greatly reduce the effects of quantization, making them almost negligible in most cases.

**Figure 11:**
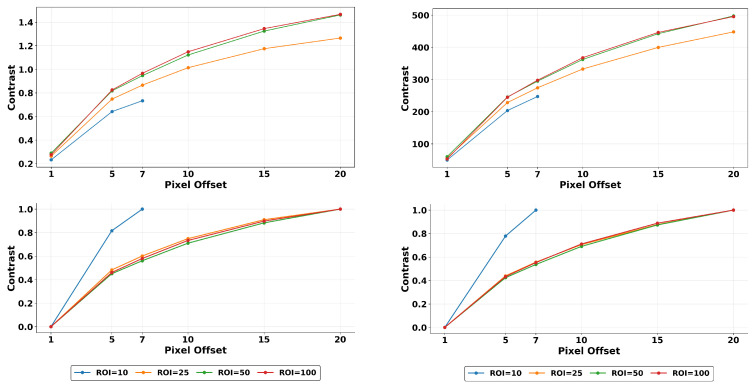
Comparison of raw magnitudes vs. multi-resolution normalization. Normalized texture features on simulated filtered images by distance offset on different quantization. (**Top**): Contrast raw magnitudes at quantization of 8 (**left**) and 128 (**right**). (**Bottom**): Contrast normalized at quantization of 8 (**left**) and 128 (**right**). Distance offset normalization in the ROIs can diminish effects of quantization as seeing when we compare the images on the left with the right. It also reduces variability caused from ROI sizes from 25 and up, increasing feature robustness.

In Figure 11 we observe that the magnitude of contrast feature exhibits significant variability due to changes in quantization levels used in estimations. However we show that the variability is reduced by orders of magnitude and as low as 2.93% for contrast features with our multi-resolution normalization. With our proposed method, on average between these quantization levels, texture variability is reduced to 7.34% while maintaining characteristic trends.

**Figure 12:**
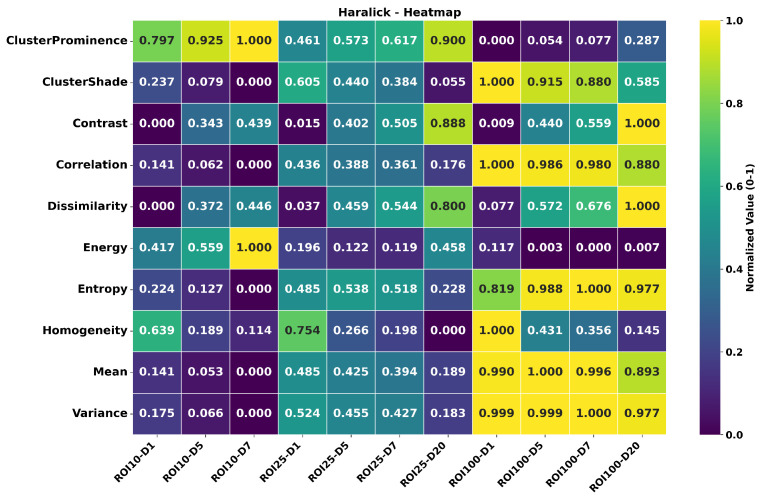
Multi-resolution normalized texture magnitudes of 10 chosen GLCM features (y-axis) calculated under different parameters combinations (x-axis). The number that follows D indicates the chosen offset and the number that follows ROI indicates ROI size. In this heatmap, we assess the effects that the parameters changes have in the magnitudes of different features.

Finally, Figure 12 presents a brief summary of the results with changing ROI and offset d for fixed quantization (here, a quantization of 32). The presented results are shown after a min-max normalization (as in Equation (2)) across each row. We display combinations of three ROI sizes and four distance offsets to highlight the magnitude changes and analyze their relative impact, which is crucial depending on the pursued task.

Analyzing ROI size, we observe that texture features characterizing order, such as entropy and energy, are most affected when using a bigger region. The larger ROI includes more information, reducing the probability of a certain pair of pixel values to appear at a given distance compared to the overall pixel pair combinations possible for that ROI. This leads to an increase in entropy and a decrease in energy. GLCM-Variance corroborates this claim, increasing rapidly compared as the ROI enlarges. Cluster shade texture magnitudes also increase as expected, given that in a bigger ROI, the probability of finding larger clusters of pixels with the same or close grayscale levels increases due to the limited pixel range, which is a direct consequence of quantization. However, structural textures contrast, homogeneity, and dissimilarity do not show much difference.

On the other hand, when analyzing changes of a given texture feature with different distances *d* and constant ROI, we observe that textures characterizing order energy, entropy, cluster shade, prominence, and tendency are not intensely affected, but structural feature contrast, homogeneity, and dissimilarity are noticeably impacted. This is expected since pixel values and numbers remain unchanged; only the approach to pair them changes. Additionally, we expect higher variations in structural features’ values the further away the elements of the pixels are due to the heterogeneous nature of the volume.

On an additional general note, the texture features (such as contrast, cluster tendency, cluster shade, and cluster prominence) with higher variations in magnitude (due to changes in quantization) employ pixel values (or higher orders of pixel value combinations) in their numerators (See Table 1 for definitions). For features like energy, entropy, and cluster prominence, higher differences due to pixel offset are seen in smaller ROIs, while higher differences due to ROI size are seen in larger pixel offsets.

### 3.6. Simulated and Clinical Data

**Figure 13:**
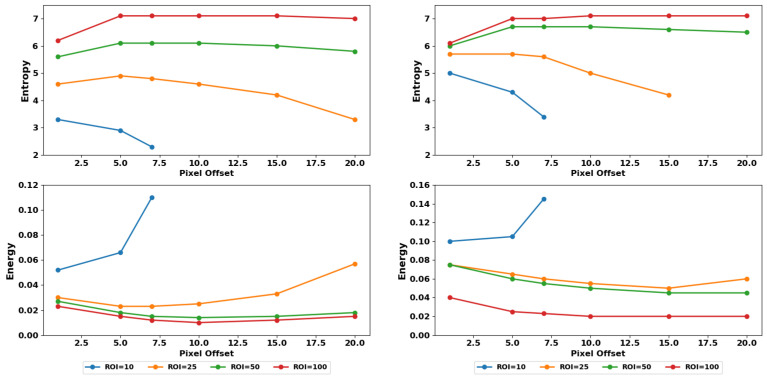
(**left**) Simulated and (**right**) clinical data texture features over different distance offsets and ROI sizes with a quantization of 32-grayscale levels. (**Top**) from **left** to **right**: simulated entropy and clinical entropy. (**Bottom**) from **left** to **right**: simulated energy and clinical energy. The similarity on trends between simulated and clinical images provide strong substantiation in the applicability of our results into clinical settings.

We examined the trends in changes due to texture magnitude variations found from simulated images against what was estimated from clinical DBT images. As mentioned in previous sections, both datasets are DBTs of 1 mm slab thickness. The acquisition angle differs, with 60° in simulated and 15° in clinical. Note that since we did not have breast density classification of clinical data, we used the average of all breast densities in simulations to compare against the clinical observations. In Figure 13, we observe that features such as entropy and energy produce similar trends between clinical and simulated data for changes in pixel offset for different ROI sizes. Note that while a new min-max normalization was described previously (Section 3.5), to reduce variabilities caused by quantization differences, we have not used that in this specific comparison as the same quantization (32-grayscale) was used for all results presented in this section. The similar trends observed from our realistic simulated DBT images vs. clinical images are encouraging and point to the validity of using simulated images to extract important information in designing robust texture analysis platforms.

## 4. Discussions and Conclusions

It is well understood that changes in imaging system parameters can impact the magnitude of texture features. In this work, we examine the effects of parameters used to estimate GLCM texture features which are widely used as radiomic metrics and AI inputs. We examined both simulated and clinical DBT images for this work. Simulated images offer ground truth, allowing the experimenter to understand the origins of variations seen in texture calculations with changes in either the imaging system or processing parameters. The textures are organized into well known classes like the following: order features, contrast features, GLCM descriptive statistical features, and cluster features (See Table 1). Here, we generally define robustness as a smaller change in texture magnitude across changes in estimation variables when calculating textures.

We show that parameters such as ROI, offset distance (d), and quantization can impact the estimated texture magnitude for even the same set of images. Among these parameters, quantization appears to have the biggest impact. Different texture features also vary at different rates with changes in these parameters. In general, features whose definitions include pixel values (or high orders of these pixel values) in their numerators show higher magnitude variations, especially due to quantization changes.

With changes in offset distance but constant ROI sizes for estimations, the textures characterizing order such as energy, entropy, cluster shade, prominence, and tendency are not intensely affected, but structural texture features such as contrast, homogeneity, and dissimilarity are noticeably impacted. For constant offset but with changes in ROI sizes, we observe that texture features characterizing order, such as entropy and energy, are most affected when using bigger ROI sizes. However structural textures such as contrast, homogeneity, and dissimilarity do not show much difference.

We further examined the results of post-processing filters like the adaptive Weiner filter as an example to show that every post-processing step employed by the vendor would impact texture calculations and magnitudes differently. Thus it would be critical to understand these behaviors when comparing images from different vendors. As an example, we show that for some texture features like contrast, the filtering generally improves the robustness (or in other words reduces the changes in texture magnitudes) with changes in ROI size selection for any given offset value. However, this is not the case with other features like cluster shade. For both of these texture features, general robustness improved with larger offset distances (above offset of 7).

The field of radiomics and AI-based imaging biomarker assessments aim to gain insights into patient disease-specific markers through estimations of texture and other features. In light of the variations due to texture estimation parameters, we examined how a specific patient-relevant change such as breast density might overlap or interact with changes in texture estimation methods. We found that texture features calculated in smaller ROIs showed significantly lower variations with changes in breast density. This factor shows that in order to extract robust and patient breast density-specific information, one has to perform texture feature calculations with larger ROI sizes. In addition, we show that some features (like cluster shade) are more sensitive to patient-specific features (like breast density) than others. We aim to examine these more carefully as part of our future work.

Through our MANOVA-ANOVA analysis we corroborate that quantization is the most influential parameter affecting GLCM features magnitudes. The significant interactions found indicate that parameter effects are not independent, and cross-study comparisons require identical parameter configurations and all these estimation parameters should be always specified. Additionally, the high $R^{2}$ values highlight the importance of standardized calculation protocols for reproducible texture analysis.

To reduce the impact of significant magnitude changes due to multiple estimation methods, we propose a new multi-resolution normalization. We show how it can mitigate the robustness issues arising from of choosing different quantization levels. We aim to test the benefits of this with clinical data from multiple vendors.

Given the widespread use of texture features as image-based radiomic features and predictive markers for risk assessment, our work offers strong caution against using them without consideration of robustness to various parameters, system configurations, or image processing changes. While radiomics-based AI models are being tested, it has been challenging to generalize them. When radiomic features demonstrate high sensitivity to parameter choices, the resulting AI models inherit this instability, potentially leading to inconsistent results. This represents a significant barrier to model generalization.

Finally, we compared some of the trends in robustness against estimation variables found from our simulated images with those found using clinical DBT images. It is encouraging to see that the trends observed from our simulated images match those using clinical data. The robustness issues we present should not diminish the potential of second-order texture features from applications in medical images once the user can understand ways to improve robustness or factors that reduce robustness. Our work can be a helpful guidance in yielding more robust calculations and understanding methods to potentially extract patient-specific information regardless of system, image processing, and texture estimation variations.

## Figures and Tables

**Table 1:** GLCM texture features.

Texture Feature	Formula
Order Features	
Energy	$\sum_{i,j}G{\left(i,j\right)}_{\theta }^{2}{,}_{d}$
Entropy	$-\sum_{i,j}G{\left(i,j\right)}_{\theta }{,}_{d}lnG{\left(i,j\right)}_{\theta }{,}_{d}$
Contrast/Structure Features	
Homogeneity	$\sum_{i,j}\frac{G{\left(i,j\right)}_{\theta }{,}_{d}}{1+|i-j|}$
Contrast	$\sum_{i,j}G{\left(i,j\right)}_{\theta }{,}_{d}{\left(i-j\right)}^{2}$
GLCM Descriptive Statistics Features	
GLCM— Mean	$\mu_{x}=\sum_{i,j}iG{\left(i,j\right)}_{\theta }{,}_{d}$
GLCM—Variance	$\sigma_{x}=\sum_{i,j}G{\left(i,j\right)}_{\theta }{,}_{d}\left(i-\mu_{x}\right)$
GLCM—Correlation	$\sum_{i,j}$ $\frac{\left(i,j\right)G{\left(i,j\right)}_{\theta }{,}_{d}-\mu_{x}\mu_{y}}{\sigma_{x}\sigma_{y}}$
Cluster Features	
Cluster—Tendency	$\sum_{i,j}{\left(i+j-\mu_{x}-\mu_{y}\right)}^{2}G{\left(i,j\right)}_{\theta }{,}_{d}$
Cluster—Shade	$\sum_{i,j}{\left(i+j-\mu_{x}-\mu_{y}\right)}^{3}G{\left(i,j\right)}_{\theta }{,}_{d}$
Cluster—Prominence	$\sum_{i,j}{\left(i+j-\mu_{x}-\mu_{y}\right)}^{4}G{\left(i,j\right)}_{\theta }{,}_{d}$

$G{\left(i,j\right)}_{\theta }{,}_{d}=$ GLCM for pixels *i* and *j*, calculated at angle θ with pixel distance *d*.

**Table 2:** Comparison of average and percentage difference for pairs of consecutive pixel offset of 5 and higher, for images with and without Wiener filter: (7-5), (10-7), (15-10), and (20-15). ROI of 10 only has the first difference (7-5) as value.

		Without Wiener Filter		With Wiener Filter	
**Texture—ROI Size**		**Avg. Change**	**Avg. Percentage Change**	**Avg. Change**	**Avg. Percentage Change**
Contrast	10	1.56	7.01%	2.50	20.72%
	25	1.99	7.11%	3.18	18.02%
	100	2.41	7.38%	3.66	19.08%
Cluster Shade	10	$-2.86\times 10^{9}$	−1.04%	$-5.15\times 10^{9}$	−1.60%
	25	$-5.19\times 10^{9}$	−1.78%	$-6.28\times 10^{9}$	−1.87%
	100	$-5.21\times 10^{9}$	−1.58%	$-5.39\times 10^{9}$	−1.46%

**Table 3:** Comparison of min-max magnitude values by ROI size between quantization grayscale levels of 8 and 128.

		Quantization—8		Quantization—128	
**Texture—ROI Size**		**Min**	**Max**	**Min**	**Max**
Contrast	10	0.18	1.09	31.52	407.48
	25	0.20	2.09	36.43	815.71
	50	0.20	2.32	38.47	868.53
	100	0.20	2.26	35.07	836.42
Cluster Shade	10	$1.66\times 10^{6}$	$3.30\times 10^{6}$	$1.66\times 10^{16}$	$3.76\times 10^{16}$
	25	$1.73\times 10^{6}$	$3.59\times 10^{6}$	$1.61\times 10^{16}$	$4.03\times 10^{16}$
	50	$2.02\times 10^{6}$	$3.95\times 10^{6}$	$1.82\times 10^{16}$	$4.38\times 10^{16}$
	100	$2.07\times 10^{6}$	$4.08\times 10^{6}$	$1.83\times 10^{16}$	$4.42\times 10^{16}$

**Table 4:** Comparison of average and percentage difference for pairs of consecutive pixel offset of 5 and higher, for breast densities of 25 and 50%: (7-5), (10-7), (15-10), and (20-15). ROI of 10 only has the first difference (7-5) as value.

		Density 25%		Density 50%	
**Texture—ROI Size**		**Avg. Change**	**Avg. Percentage Change**	**Avg. Change**	**Avg. Percentage Change**
Contrast	10	1.981	17.75%	3.018	23.29%
	25	2.095	16.42%	3.300	22.88%
	100	2.528	18.46%	3.691	23.82%
Cluster Shade	10	$-5.18\times 10^{9}$	−1.73%	$-5.13\times 10^{9}$	−1.49%
	25	$-4.57\times 10^{9}$	−1.40%	$-2.80\times 10^{9}$	−0.77%
	100	$-2.36\times 10^{9}$	−0.65%	$-2.18\times 10^{9}$	−0.56%

**Table 5:** MANOVA main effects analysis: explained variance and effect significance. Quantization is the parameter with most significant effect across all features.

Feature	$R^{2}$	ROI	Distance	Quantization
		F-Value	F-Value	F-Value
Energy	0.906	2685.14 *	71.45 *	48,474.57 *
Entropy	0.921	22,500.12 *	266.16 *	38,692.21 *
Homogeneity	0.971	4135.10 *	12,421.33 *	154,412.77 *
Contrast	0.759	27.37 *	1227.18 *	14,536.80 *
Mean	0.962	2126.72 *	44.69 *	134,999.03 *
Correlation	0.987	3763.39 *	82.09 *	397,647.75 *

Note: Results marked with an asterisk (*) are statistically significant at *p* < 0.01. All features showed 100% effect significance across parameters.

**Table 6:** Interaction effects: model performance and significant interactions. Both 2-way and 3-way interactions improved model performance.

Feature	2-Way $R^{2}$	3-Way $R^{2}$	ROI × D	ROI × Qt	D × Qt	3-Way
Energy	0.948	0.950	*	*	*	*
Entropy	0.991	0.993	*	*	*	*
Homogeneity	0.988	0.988	*	*	*	*
Contrast	0.917	0.917	*	*	*	*
Mean	0.972	0.972	*	*	NS	*
Correlation	0.998	0.998	*	*	*	*

Note: Results marked with (*) are statistically significant and with (NS) not significant at *p* < 0.01.

## Data Availability

The data that support the findings of this study are available from the corresponding author upon reasonable request.

## Abbreviations

The following abbreviations are used in this manuscript:

DBT	Digital Breast Tomosynthesis
GLCM	Gray Level Co-occurrence Matrix
ROI	Region of Interest
VOI	Volume of Interest
